# The Use of Repetitive Transcranial Magnetic Stimulation to Improve Cognitive Impairment in Patients With Stroke Based on rs-fMRI Findings: Protocol for a Meta-Analysis

**DOI:** 10.2196/77931

**Published:** 2025-10-02

**Authors:** Xin Xiang, Hao Li, Lin Lu, Yuting Cao, Chunzhen Li, Lubo Xiao, Furong Liu, Yi Ran, Hong Zhang, Ning Zhao

**Affiliations:** 1 College of Acupuncture & Tuina and Rehabilitation Hunan University of Chinese Medicine Changsha China; 2 Department of Rehabilitation Shenzhen Nanshan People's Hospital/Affiliated Nanshan Hospital of Shenzhen University Shenzhen China; 3 School of Clinical Medicine Ningxia Medical University Yinchuan China

**Keywords:** repetitive transcranial magnetic stimulation, stroke, cognitive impairment, resting-state functional magnetic resonance imaging, meta-analysis, protocol

## Abstract

**Background:**

Poststroke cognitive impairment (PSCI) is a chronic form of poststroke cognitive dysfunction that affects approximately one-third of the survivors of stroke. PSCI significantly increases the rates of mortality and functional disabilities, such as limitations in motor function, speech, and activities of daily living. Therefore, effective treatments are needed for patients with PSCI. Repetitive transcranial magnetic stimulation (rTMS) has been shown to exert beneficial behavioral effects in patients with PSCI. More importantly, a limited number of neuroimaging studies with small sample sizes have reported the beneficial effects of rTMS on brain plasticity and its reciprocal influence on cognitive and behavioral performance. Resting-state functional magnetic resonance imaging (rs-fMRI) has been widely used to study changes in brain activity, but there is no consensus regarding which brain regions play pivotal roles in rTMS for patients with PSCI.

**Objective:**

This study aims to explore the therapeutic effects of rTMS on changes in the brain activity of patients with PSCI, thereby providing robust evidence to elucidate its neuroimaging mechanisms.

**Methods:**

In this meta-analysis, we will systematically search the PubMed, Embase, Cochrane Library, Web of Science, China Biology Medicine, and China National Knowledge Infrastructure databases, VIP Chinese Science and Technology Periodical Database, and the China WanFang Database up to December 2024 to identify randomized controlled trials comparing active rTMS with sham stimulation conditions or conventional control conditions in patients with PSCI. The primary outcomes will include the amplitude of low-frequency fluctuation, fractional amplitude of low-frequency fluctuation, regional homogeneity, and functional connectivity across the whole brain. The secondary outcomes will include the Montreal Cognitive Assessment and Mini-Mental State Examination scores. Statistical analyses will be conducted via Review Manager (version 5.4), Seed-based d Mapping with Permutation of Subject Images (version 6.23), and Stata (version 18.0) software to assess study quality, evaluate the risk of bias, and analyze the outcome measures.

**Results:**

The study will offer a comprehensive analysis of the available evidence on the use of rTMS to improve cognitive impairment in patients with stroke based on rs-fMRI findings. The meta-analysis will be conducted from July 2024 to April 2026, following this predefined protocol. The process encompasses database searching and study screening (to be concluded by October 2025), data extraction and synthesis (to be completed by December 2025), and subsequent manuscript preparation and submission (anticipated by April 2026).

**Conclusions:**

This meta-analysis will provide insights into the therapeutic potential of rTMS to improve cognitive impairment in patients with stroke. It will also highlight the strengths and limitations of the existing literature and suggest directions for future research. Ultimately, our study may aid future clinical decision-making concerning PSCI rehabilitation programs and provide evidence-based medical insights into the neuroimaging mechanisms of rTMS treatment for PSCI.

**International Registered Report Identifier (IRRID):**

DERR1-10.2196/77931

## Introduction

### Background

Stroke is a serious neurological disease. According to the 2022 release of the *China Stroke Report*, the incidence rate of stroke in China is 2022.0 per 100,000, with an annual incidence of 276.7 per 100,000 and a mortality rate of 153.9 per 100,000 [1]. Poststroke cognitive impairment (PSCI) is defined as a sustained cognitive deficit for at least 6 months after a stroke [2], which affects about one-third of patients with stroke [3]. PSCI significantly elevates mortality risk [4], impedes the recovery of motor and speech functions and activities of daily living [5], and substantially increases the caregiver burden and socioeconomic costs [6]. Therefore, focusing on the rehabilitation of patients with PSCI is of crucial clinical importance.

Repetitive transcranial magnetic stimulation (rTMS) is a safe, efficient, and well-tolerated noninvasive neuromodulation technique [7]. It induces cortical excitability changes through electromagnetic induction [8]. For PSCI, 2 primary therapeutic models exist: the interhemispheric inhibition model and the compensation model. The interhemispheric inhibition model posits that stroke disrupts callosal balance, suppressing the affected hemisphere while hyperactivating the unaffected hemisphere [9]. rTMS counteracts this by upregulating the affected hemisphere excitability (via high-frequency stimulation) or downregulating the unaffected hemisphere activity (via low-frequency stimulation) [10]. The compensation model proposes neuroplastic reorganization around lesions or in contralesional regions to compensate for deficits [11]. These models are integrated in the bimodal balance-recovery model, which recommends selecting rTMS strategies based on the structural preservation in the affected hemisphere [12].

Studies indicate that rTMS can modulate neural activity in specific brain regions [13,14] and improve PSCI behavioral indicators [15-23]. Numerous meta-analyses have consistently supported the effectiveness of rTMS intervention for improving cognitive function in patients with stroke [15-23]. Multiple meta-analyses consistently demonstrate rTMS-induced cognitive improvement in patients after stroke across diverse assessment tools (the Montreal Cognitive Assessment [MoCA], the Mini-Mental State Examination [MMSE], the Loewenstein Occupational Therapy Cognitive Assessment, and the Repeatable Battery for the Assessment of Neuropsychological Status) [16,17]. Significant global cognitive benefits have been observed with both high-frequency rTMS (HF-rTMS) and low-frequency rTMS (LF-rTMS) protocols targeting the dorsolateral prefrontal cortex (DLPFC) [16,17]. Enhanced executive function and working memory (although not memory or attention) have also been observed when rTMS is combined with cognitive training, with optimal effects from HF-rTMS over the left DLPFC or LF-rTMS over the right DLPFC [18]. The superior efficacy of HF-rTMS compared with LF-rTMS for DLPFC stimulation in improving cognition and functional independence (as measured by the Modified Barthel Index) has also been reported [19]. Although these studies have confirmed the behavioral efficacy of rTMS in improving PSCI, the potential neuroimaging mechanisms underlying this behavioral effect remain unclear.

Resting-state functional magnetic resonance imaging (rs-fMRI) provides a noninvasive tool for mapping rTMS-induced neural changes in PSCI [24,25]. Evidence indicates that rTMS can modulate the excitability and functional connectivity (FC) in cognition-related networks (eg, enhanced DLPFC-frontotemporal coupling [26], and increased amplitude of low-frequency fluctuation [ALFF] in the medial prefrontal cortex [27]). Concurrently, cognitive scores (MoCA, MMSE, and Modified Barthel Index) and neural activity have also improved [26-31]. However, systematic evidence quantifying the consistent neuroimaging effects of rTMS remains limited, which hinders the clarification of its mechanistic pathways and clinical optimization. Existing findings are predominantly derived from small-sample, single-center randomized controlled trials (RCTs), resulting in insufficient statistical power and generalizability [27,28]. Although narrative reviews acknowledge the potential of functional magnetic resonance imaging (fMRI) for elucidating rTMS mechanisms, they rely on the qualitative synthesis of methodologically heterogeneous studies and cannot integrate spatial patterns of neural change [32]. Crucially, no quantitative meta-analysis has yet synthesized coordinate-based neuroimaging data to identify robust, spatially convergent neural targets of rTMS in PSCI.

### Objectives

This coordinate-based meta-analysis (CBMA) aims to (1) quantitatively synthesize evidence regarding the effects of rTMS on cognitive function in patients with PSCI, (2) identify consistent patterns of rTMS-induced neural changes using voxel-wise meta-analysis of fMRI data, (3) explore the effects of intervention parameters—including stimulation frequency, target location, and intensity—as potential moderators of efficacy, and (4) explore whether rTMS sham stimulation intervention has a placebo effect. This study will pioneer the application of CBMA in rTMS neuromodulation research for PSCI, with the goal of advancing mechanistic understanding and supporting evidence-based optimization of stimulation protocols.

## Methods

The protocol of this meta-analysis will be conducted and reported in accordance with the Preferred Reporting Items for Systematic Reviews and Meta-Analysis Protocols (PRISMA-P) statement guidelines [33]. The PRISMA-P checklist is shown in Multimedia Appendix 1. This meta-analysis protocol was registered in PROSPERO (CRD42024562477).

### Study Inclusion and Exclusion Criteria

#### Types of Studies

This meta-analysis will include RCTs reporting rTMS interventions based on rs-fMRI for improving PSCI. Studies comparing rTMS with conventional treatments or sham stimulation will be eligible. All eligible rs-fMRI studies that meet these criteria will be included, regardless of language or publication type. Non-RCTs, such as case reports, conference papers, animal experiments, systematic reviews, and meta-analyses, will be excluded.

#### Types of Participants

The participants in the study will consist of patients with PSCI, with inclusion criteria independent of sex, nationality, ethnicity, or treatment setting.

#### Types of Interventions

##### Interventions

We will consider trials where rTMS is administered for at least 1 session. Interventions include HF-rTMS, LF-rTMS, intermittent theta burst stimulation, continuous theta burst stimulation, and single-pulse stimulation, or comparisons among these 5 rTMS modalities.

##### Comparators

The control group will receive conventional treatments or sham stimulation.

##### Combination Interventions

We plan to include combination interventions where rTMS treatment is combined with pharmacological treatment or other clinical intervention methods. We will only include trials where both the intervention and control groups receive identical pharmacological treatments or other clinical intervention methods.

#### Outcome Measures

##### Primary Outcomes

Studies reporting any fMRI analysis method capable of effectively assessing changes in brain activity and functional states, such as the ALFF, fractional ALFF (fALFF), regional homogeneity (ReHo), and FC across the whole brain, will be considered eligible for inclusion. The ALFF and fALFF are validated rs-fMRI analysis methods that evaluate neuronal activity by measuring low-frequency oscillations (0.01-0.08 Hz) in the blood oxygen level–dependent (BOLD) signal [34]. The ALFF and fALFF accurately reflect spontaneous neuronal activity in the resting state from an energy perspective; increased ALFF and fALFF indicate heightened spontaneous activity within a brain region, whereas decreased ALFF and fALFF suggest reduced spontaneous activity [35]. ReHo assesses regional synchronization of BOLD signal fluctuations based on local correlations [36]. This analysis method provides stable insights into the temporal coherence of local neuronal activity. FC is defined as the temporal correlation between neurophysiological (functional) measurements obtained from distinct brain regions [37]. FC can reveal the key roles and regulatory influences on the activity of the target brain area of the whole network.

These methods can be used to explore the characteristics and patterns of changes in brain activity in patients with PSCI after rTMS treatment, revealing their neuroimaging mechanisms. All evaluations are conducted via fMRI and reported in the standard stereotaxic space of Talairach or the Montreal Neurological Institute with 3D coordinates (x, y, and z).

##### Secondary Outcomes

Any study reporting an assessment scale that can effectively reflect cognitive function in patients with PSCI, such as the MoCA and MMSE, will be considered eligible. Both the MoCA score and the MMSE score have been shown to correlate with age and years of education. The MoCA is a simple, stand-alone cognitive screening tool known for its high sensitivity. It assesses important cognitive domains and is a 30-point test. It also has excellent test-retest reliability [38,39]. The MMSE is a concise, standardized assessment tool used to evaluate patients’ cognitive status. The maximum score is 30 points. It measures orientation, attention, immediate and short-term memory, language, and the ability to follow basic verbal and written instructions. The test yields a cumulative score that indicates an individual’s level of cognitive functioning on a standardized scale [39,40].

### Search Methods for the Identification of Studies

#### Electronic Searches

Our search will include databases such as the PubMed, Embase, Cochrane Library, Web of Science, China Biology Medicine, and China National Knowledge Infrastructure databases, the VIP Chinese Science and Technology Periodical Database, and the China WanFang Database from their inception up to December 31, 2024. Our search strategy will incorporate key terms such as “stroke,” “transcranial magnetic stimulation,” “cognition,” and “magnetic resonance imaging.” To ensure comprehensiveness, we will conduct an exhaustive search within each database across all relevant fields (eg, titles, abstracts, and keywords) using both Medical Subject Headings (MeSH) and free-text terms. Taking the PubMed database as an example, the retrieval strategy is shown in Table 1. The search strategies for all the databases are described in detail in Multimedia Appendix 2.

**Table 1:** Search strategy for PubMed.

Number	Terms
1	Stroke [MeSH^a^ Terms]
2	Strokes OR Cerebrovascular Accident OR Cerebrovascular Apoplexy OR Cerebrovascular Stroke OR Cerebral Infarction OR Cerebral Hemorrhage
3	(Stroke [MeSH Terms]) OR (Strokes OR Cerebrovascular Accident OR Cerebrovascular Apoplexy OR Cerebrovascular Stroke OR Cerebral Infarction OR Cerebral Hemorrhage)
4	Transcranial Magnetic Stimulation [MeSH Terms]
5	Transcranial Magnetic Stimulations OR Repetitive Transcranial Magnetic Stimulation OR rTMS^b^ OR Intermittent Theta Burst Stimulation OR Continuous Theta Burst Stimulation
6	(Transcranial Magnetic Stimulation [MeSH Terms]) OR (Transcranial Magnetic Stimulations OR Repetitive Transcranial Magnetic Stimulation OR rTMS OR Intermittent Theta Burst Stimulation OR Continuous Theta Burst Stimulation)
7	Cognition [MeSH Terms]
8	Cognitive Dysfunction [MeSH Terms]
9	Cognition OR Cognitive Dysfunction OR Cognitive Impairment
10	((Cognition [MeSH Terms]) OR (Cognitive Dysfunction [MeSH Terms])) OR (Cognition OR Cognitive Dysfunction OR Cognitive Impairment)
11	Magnetic Resonance Imaging [MeSH Terms]
12	Magnetic Resonance Imaging OR Functional Magnetic Resonance Imaging OR fMRI^c^ OR Resting-state Functional Magnetic Resonance Imaging OR rs-fMRI^d^
13	(Magnetic Resonance Imaging [MeSH Terms]) OR (Magnetic Resonance Imaging OR Functional Magnetic Resonance Imaging OR fMRI OR Resting-state Functional Magnetic Resonance Imaging OR rs-fMRI)
14	(((((Stroke [MeSH Terms]) OR (Strokes OR Cerebrovascular Accident OR Cerebrovascular Apoplexy OR Cerebrovascular Stroke OR Cerebral Infarction OR Cerebral Hemorrhage)) AND ((Transcranial Magnetic Stimulation [MeSH Terms]) OR (Transcranial Magnetic Stimulations OR Repetitive Transcranial Magnetic Stimulation OR rTMS OR Intermittent Theta Burst Stimulation OR Continuous Theta Burst Stimulation))) AND (((Cognition [MeSH Terms]) OR (Cognitive Dysfunction [MeSH Terms])) OR (Cognition OR Cognitive Dysfunction OR Cognitive Impairment))) AND ((Magnetic Resonance Imaging [MeSH Terms]) OR (Magnetic Resonance Imaging OR Functional Magnetic Resonance Imaging OR fMRI OR Resting-state Functional Magnetic Resonance Imaging OR rs-fMRI))) AND ((1879/01/01 [Date - Publication] : 2024/12/31 [Date - Publication]))

^a^MeSH: Medical Subject Headings.

^b^rTMS: repetitive transcranial magnetic stimulation.

^c^fMRI: functional magnetic resonance imaging.

^d^rs-fMRI: resting-state functional magnetic resonance imaging.

#### Reference Lists

We will examine the reference lists of all included trials.

### Data Collection and Management

#### Selection of Studies

**Figure 1:**
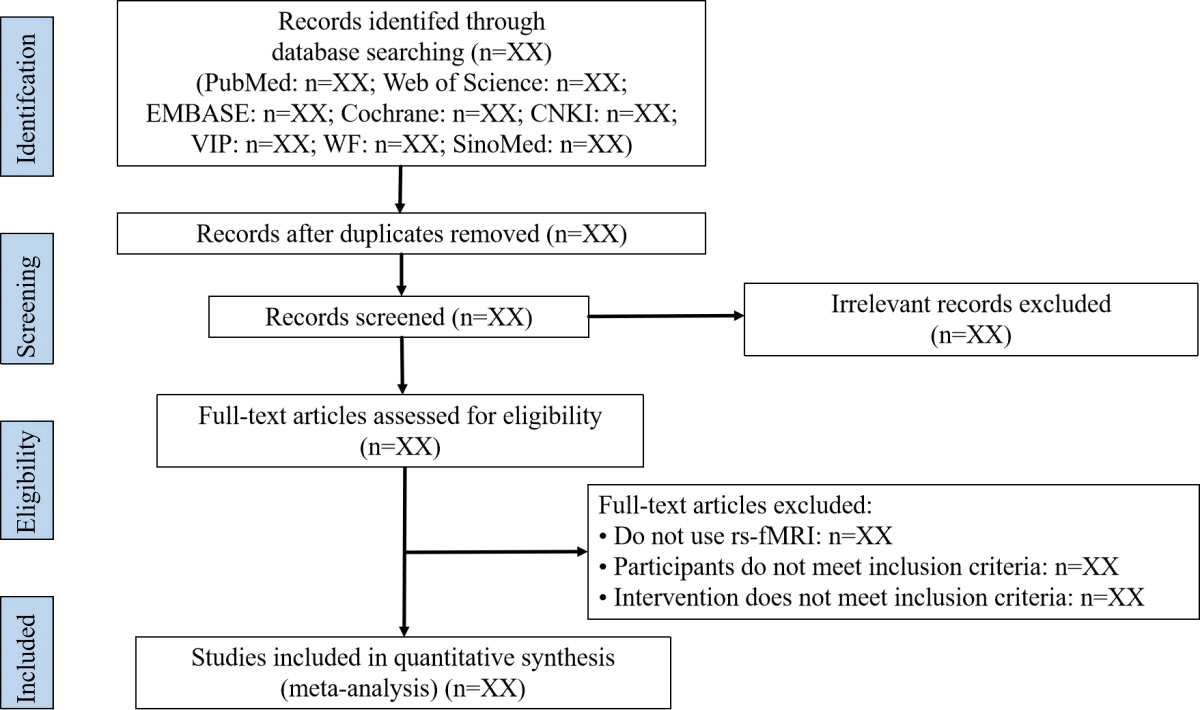
Literature screening process.

The RCTs included will be reviewed by 2 independent researchers (XX and LX). NoteExpress software (version 4.0.0; Beijing Aegean Music Technology) will be used for independent management of the search results from the aforementioned databases. Initially, duplicate publications will be removed. After reviewing titles and abstracts, studies that do not meet the criteria will be excluded. Finally, the full texts of the remaining studies will be downloaded and reviewed to exclude those that do not meet the criteria. Any discrepancies related to study selection outcomes will be discussed and resolved by a third researcher (NZ) following cross-checking. The flowchart detailing all the study screening procedures is shown in Figure 1.

#### Data Extraction

Two researchers (XX and FL) will independently extract data according to the designed data extraction table. To ensure the consistency of the data extraction process, 2 researchers will be specially trained in advance. Disagreements will be resolved by consulting a third researcher (NZ). The basic information to be extracted will include the following: the essential characteristics of the included literature (first author, publication year, sample size, duration of illness, age, sex, intervention measures, fMRI analysis methods, and secondary outcome measures); specific parameters and treatment protocols of rTMS (stimulation frequency, stimulation intensity, stimulation site, total pulse number, coil type, and duration); and fMRI-related information (peak coordinates, effect sizes [*t* test values, or equivalent *z* scores or *P* values], analysis software, and standard space).

#### Quality Assessment

On the basis of the assessment approaches adopted in prior neuroimaging meta-analyses [41,42], the methodological quality of the included fMRI studies will be evaluated using a modified checklist. This tool comprises 2 categories and 13 items that assess key aspects such as sample size, methodological rigor, and reporting completeness, with a total possible score of 20 points. Higher scores indicate superior methodological quality. The checklist is shown in Multimedia Appendix 3. In addition, 2 researchers (XX and YR) will independently use the refined risk of bias in randomized trials tool to systematically evaluate the risk of bias across specific domains: bias arising from the randomization process, bias due to deviations from intended interventions, bias due to missing outcome data, bias in the measurement of the outcome, and bias in the selection of the reported result. The assessment of each domain will be rated as “low risk,” “some concerns,” or “high risk” [43]. Assessments will be conducted independently and then cross-checked. Disagreements will be resolved by consulting a third researcher (NZ).

#### Data Synthesis and Meta-Analysis

The outcome measures will be statistically analyzed using the following software: Seed-based d Mapping with Permutation of Subject Images (SDM-PSI; version 6.23) and Stata (version 18.0; StataCorp).

#### Imaging Data

The SDM-PSI will be used to analyze differences in brain activity between groups and within groups. Initially, peak coordinates and effect sizes (*t* test values, equivalent *z* scores or *P* values) from each study report will be extracted to prepare text files of peak values. During preprocessing, the software will use these files to recreate the effect size CIs within studies. A meta-analysis will then be conducted to determine the mean effects of ALFF, fALFF, ReHo, and FC coordinates, weighted by sample size and variance, for each study. Finally, a meta-regression analysis will be conducted to identify relationships between ALFF, fALFF, ReHo, and FC changes and MoCA, MMSE, age, and course of disease.

#### Behavioral Data

Stata software will be used to analyze behavioral data.

#### Continuous Outcomes

For outcomes assessed using the same scale, we will summarize the data with mean differences and 95% CIs. For outcomes assessed using different but conceptually similar scales, we will use standardized mean differences and 95% CIs to summarize the data. Forest plots will be generated to display the hypothesis test results.

#### Dichotomous Outcomes

We will summarize dichotomous outcomes using odds ratios, risk ratios, or risk differences, along with their corresponding 95% CIs.

#### Dealing With Missing Data

We will contact the study authors to obtain the missing data. If we cannot obtain these data, we will directly report the outcomes of these studies rather than include them in the meta-analysis.

#### Assessment of Heterogeneity

The methodological heterogeneity inherent in fMRI studies presents a challenge for meta-analytic synthesis. To evaluate between-study heterogeneity, we will extract peak coordinates reported in the Montreal Neurological Institute space and quantify statistical inconsistency using the *I*^2^ statistic [44]. An *I*^2^ value <50% will be interpreted as indicating low heterogeneity. For behavioral data, depending on trial homogeneity (*P*>.10, *I*^2^<50%), fixed-effects or random-effects models will be used for data synthesis. If high levels of heterogeneity are indicated by visual inspection of the forest plots or an *I*^2^ statistic of ≥50%, we will explore the sources of heterogeneity via predefined subgroup analyses or sensitivity analyses.

#### Assessment of Reporting Bias

Funnel plots will be used to assess publication bias for outcomes that are reported in more than 10 studies. Symmetrical funnels will indicate no publication bias, whereas asymmetry will suggest its presence. For outcomes that are reported in fewer than 10 studies, the Egger test will be used to assess publication bias, and the *P* value will be reported accordingly.

#### Analysis of Subgroups

We will perform subgroup analysis according to the following hierarchy: tier 1 (effect modifiers)—intervention strategies (pure rTMS interventions or combination interventions) and type of control intervention (sham rTMS or non-rTMS); tier 2 (stimulation parameters)—stimulation site (DLPFC or non-DLPFC), frequency (low frequency or high frequency), intensity (%Motor Threshold) and pattern (HF-rTMS, LF-rTMS, intermittent theta burst stimulation, continuous theta burst stimulation, or single-pulse stimulation); and tier 3 (clinical characteristics)—age (<60 y or ≥60 y), sex (male or female), disease course (acute: <3 mo, subacute: 3-6 mo or chronic: >6 mo), hemiplegic side (left, right or bilateral), type of stroke (ischemic or hemorrhagic), and lesion location (cortical, subcortical or mixed).

This study will also evaluate the difference in efficacy between pure rTMS interventions and combination interventions using a subgroup interaction test (*P*<.05 indicates significant interaction), and calculate the *E* value to quantify how strong residual confounding would need to be to overturn the conclusion (*E* value >2.0 indicates robustness).

These analyses aim to (1) identify potential effect modifiers contributing to heterogeneity, (2) quantify the magnitude of effect size variations across clinically relevant subgroups, (3) delineate optimal stimulation parameters for specific patient phenotypes, and (4) assess the robustness of primary outcomes by controlling for confounding variables.

#### Sensitivity Analysis

We will investigate the robustness of our findings using sensitivity analysis. Specifically, we will apply the leave-one-out method. This process involves systematically excluding specific studies to assess their individual impact on the results. We will evaluate the influence of bias risk (excluding studies with high bias risk), data synthesis methodology, and significant heterogeneity (excluding trials identified as major sources of heterogeneity).

### Grading of Recommendations Assessment, Development, and Evaluation Assessment

The certainty of the evidence will be evaluated using the Grading of Recommendations Assessment, Development, and Evaluation (GRADE) framework [45]. This systematic approach assigns 1 of 4 grades, “high,” “moderate,” “low,” or “very low,” to each outcome, considering limitations in design and implementation, indirectness of evidence, unexplained heterogeneity, imprecision of results, and high probability of reporting bias [46]. Two independent researchers (XX and YR) will use the “GRADEprofiler” software to assess the evidence and then import the findings into the Review Manager software (version 5.4; The Nordic Cochrane Centre, The Cochrane Collaboration). In case of discrepancies between the researchers’ assessments, a third researcher (NZ) will arbitrate.

The GRADE framework was originally designed to assess the quality of evidence for clinical outcomes (eg, mortality, symptom severity, and functional status) and is not directly applicable to neuroimaging-derived biomarkers such as ALFF, ReHo, and FC, which serve as mechanistic or surrogate end points. Therefore, in this study, the GRADE tool will be applied exclusively to behavioral measures (eg, MoCA and MMSE) for evaluating the certainty of evidence.

## Results

The results of this meta-analysis will offer a comprehensive analysis of the available evidence on the use of rTMS to improve cognitive impairment in patients with stroke based on rs-fMRI findings. The meta-analysis process began with protocol development and registration in July 2024. A structured literature search and database screening will follow. Title and abstract screening, along with full-text review and eligibility assessment, will be completed by October 2025. Data extraction, synthesis, and analysis of findings will be completed by December 2025. Manuscript drafting will begin in January 2026, and the final review and submission of the completed meta-analysis are anticipated by April 2026.

## Discussion

### Anticipated Findings

The study aims to investigate how rTMS improves cognitive function in patients with PSCI at the neuroimaging level. We plan to integrate relevant studies and introduce CBMA to quantitatively synthesize findings from individual neuroimaging studies [47]. In this study, we will apply SDM-PSI, which is a new algorithm for CBMA used for analyzing rs-fMRI imaging data [48,49]. First, we will use CBMA to explore patterns of neuroimaging biomarker changes in patients with PSCI following rTMS intervention. Second, we will use CBMA maps to compare differences in brain region changes before and after interventions and between groups. Third, we will use meta-regression analysis methods to investigate correlations between changes in neuroimaging indicators in different brain regions and behavioral and demographic variables. The importance of this study is that it will provide intuitive and visual imaging evidence for the efficacy of rTMS and may aid in clinical decision-making regarding PSCI rehabilitation programs through CBMA.

### Limitations of This Study Design

A potential limitation of this study is that studies using rTMS for the treatment of animals were excluded from our meta-analysis. In addition, our study only reports imaging metrics such as ALFF, fALFF, ReHo, and FC. Other imaging metrics from rs-fMRI, such as effective connectivity and small-world properties, were not considered. Imaging metrics that reflect white matter and gray matter structure, such as diffusion tensor imaging and voxel-based morphometry, were also excluded. Moreover, the field of rs-fMRI still faces challenges. First, temporal variability includes susceptibility to physiological noise (eg, respiratory and cardiac artifacts) and state-dependent fluctuations [50]. Second, interpretive ambiguity arises from difficulty in distinguishing neural from vascular contributions to the BOLD signal [51]. Third, analytical heterogeneity results from issues with cross-study comparability arising from inconsistent preprocessing pipelines and connectivity metrics [52]. These limitations need to be addressed in future related studies.

### Conclusions

Our protocol, based on rs-fMRI findings, will summarize the beneficial role of neuroimaging metric changes after rTMS intervention in cognitive-related brain regions compared with the baseline level and the control conditions. Moreover, this meta-analysis will investigate the relationship between neuroimaging changes and behavioral outcomes. In summary, our study may aid future clinical decision-making concerning PSCI rehabilitation programs and provide evidence-based medical insights into the neuroimaging mechanisms of rTMS treatment for PSCI.

## Appendix

Multimedia Appendix 1 PRISMA-P (Preferred Reporting Items for Systematic Review and Meta-Analysis Protocols) 2015 checklist: recommended items to address in a systematic review protocol.

Multimedia Appendix 2 Search strategy.

Multimedia Appendix 3 Criteria for objective assessment of the methodological quality of individual studies.

## Abbreviations

ALFF: amplitude of low-frequency fluctuation
BOLD: blood oxygen level–dependent
CBMA: coordinate-based meta-analysis
DLPFC: dorsolateral prefrontal cortex
fALFF: fractional ALFF
FC: functional connectivity
GRADE: Grading of Recommendations Assessment, Development, and Evaluation
HF-rTMS: high-frequency repetitive transcranial magnetic stimulation
LF-rTMS: low-frequency repetitive transcranial magnetic stimulation
MeSH: Medical Subject Headings
MMSE: Mini-Mental State Examination
MoCA: Montreal Cognitive Assessment
PSCI: poststroke cognitive impairment
RCT: randomized controlled trial
ReHo: regional homogeneity
rs-fMRI: resting-state functional magnetic resonance imaging
rTMS: repetitive transcranial magnetic stimulation
SDM-PSI: Seed-based d Mapping with Permutation of Subject Images

## Data Availability

Data sharing is not applicable to this article as no data sets were generated or analyzed during this study.
