# Supplementary material for: The Use of Repetitive Transcranial Magnetic Stimulation to Improve Cognitive Impairment in Patients With Stroke Based on rs-fMRI Findings: Protocol for a Meta-Analysis
Source: JMIR Res Protoc. 2025 Oct 2;14:e77931. doi: 10.2196/77931 (PMC12531584; doi:10.2196/77931)
Supplement: Multimedia Appendix 2 [file resprot_v14i1e77931_app2.docx]

**Multimedia Appendix 2. Search strategy**

**Search strategy for PubMed**

| Number | Terms |
| --- | --- |
| #1 | “Stroke”[Mesh] |
| #2 | (((((((((((((((((((((((((((Strokes) OR (Cerebrovascular Accident)) OR (Cerebrovascular Accidents)) OR (CVA)) OR (CVAs)) OR (Cerebrovascular Apoplexy)) OR (Apoplexy, Cerebrovascular)) OR (Vascular Accident, Brain)) OR (Brain Vascular Accident)) OR (Brain Vascular Accidents)) OR (Vascular Accidents, Brain)) OR (Cerebrovascular Stroke)) OR (Cerebrovascular Strokes)) OR (Stroke, Cerebrovascular)) OR (Strokes, Cerebrovascular)) OR (Apoplexy)) OR (Cerebral Stroke)) OR (Cerebral Strokes)) OR (Stroke, Cerebral)) OR (Strokes, Cerebral)) OR (Stroke, Acute)) OR (Acute Stroke)) OR (Acute Strokes)) OR (Strokes, Acute)) OR (Cerebrovascular Accident, Acute)) OR (Acute Cerebrovascular Accident)) OR (Acute Cerebrovascular Accidents)) OR (Cerebrovascular Accidents, Acute) |
| #3 | “Cerebral Infarction”[Mesh] |
| #4 | ((((((((((((((((((((((((Cerebral Infarctions) OR (Infarctions, Cerebral)) OR (Infarction, Cerebral)) OR (Cerebral Infarct)) OR (Cerebral Infarcts)) OR (Infarct, Cerebral)) OR (Infarcts, Cerebral)) OR (Cerebral Infarction, Left Hemisphere)) OR (Left Hemisphere, Infarction, Cerebral)) OR (Infarction, Left Hemisphere, Cerebral)) OR (Left Hemisphere, Cerebral Infarction)) OR (Cerebral, Left Hemisphere, Infarction)) OR (Infarction, Cerebral, Left Hemisphere)) OR (Subcortical Infarction)) OR (Infarction, Subcortical)) OR (Infarctions, Subcortical)) OR (Subcortical Infarctions)) OR (Posterior Choroidal Artery Infarction)) OR (Anterior Choroidal Artery Infarction)) OR (Cerebral Infarction, Right Hemisphere)) OR (Right Hemisphere, Cerebral Infarction)) OR (Infarction, Right Hemisphere, Cerebral)) OR (Right Hemisphere, Infarction, Cerebral)) OR (Cerebral, Right Hemisphere, Infarction)) OR (Infarction, Cerebral, Right Hemisphere) |
| #5 | “Cerebral Hemorrhage”[Mesh] |
| #6 | ((((((((((((((((((((((Hemorrhage, Cerebrum) OR (Cerebrum Hemorrhage)) OR (Cerebrum Hemorrhages)) OR (Hemorrhages, Cerebrum)) OR (Cerebral Parenchymal Hemorrhage)) OR (Cerebral Parenchymal Hemorrhages)) OR (Hemorrhage, Cerebral Parenchymal)) OR (Hemorrhages, Cerebral Parenchymal)) OR (Parenchymal Hemorrhage, Cerebral)) OR (Parenchymal Hemorrhages, Cerebral)) OR (Intracerebral Hemorrhage)) OR (Hemorrhage, Intracerebral)) OR (Hemorrhages, Intracerebral)) OR (Intracerebral Hemorrhages)) OR (Hemorrhage, Cerebral)) OR (Cerebral Hemorrhages)) OR (Hemorrhages, Cerebral)) OR (Brain Hemorrhage, Cerebral)) OR (Brain Hemorrhages, Cerebral)) OR (Cerebral Brain Hemorrhage)) OR (Cerebral Brain Hemorrhages)) OR (Hemorrhage, Cerebral Brain)) OR (Hemorrhages, Cerebral Brain) |
| #7 | #1 OR #2 OR #3 OR #4 OR #5 OR #6 |
| #8 | “Transcranial Magnetic Stimulation”[Mesh] |
| #9 | (((((((((((((((((Magnetic Stimulation, Transcranial) OR (Magnetic Stimulations, Transcranial)) OR (Stimulation, Transcranial Magnetic)) OR (Stimulations, Transcranial Magnetic)) OR (Transcranial Magnetic Stimulations)) OR (Transcranial Magnetic Stimulation, Single Pulse)) OR (Transcranial Magnetic Stimulation, Paired Pulse)) OR (Transcranial Magnetic Stimulation, Repetitive)) OR (rTMS)) OR (HF-rTMS)) OR (LF-rTMS)) OR (High Frequency-rTMS)) OR (Low Frequency-rTMS)) OR (Intermittent Theta Burst Stimulation)) OR (iTBS)) OR (Continuous Theta Burst Stimulation)) OR (cTBS)) OR (Non-invasive) |
| #10 | #8 OR #9 |
| #11 | “Cognition”[Mesh] |
| #12 | “Cognitive Dysfunction”[Mesh] |
| #13 | (((((((Cognition) OR (Cognitive Dysfunction)) OR (Cognitive Impairment)) OR (Memory)) OR (Attention)) OR (Language)) OR (Executive function)) OR (Visuospatial function) |
| #14 | #11 OR #12 OR #13 |
| #15 | “Magnetic Resonance Imaging”[Mesh] |
| #16 | (((((((((((Magnetic Resonance Imaging) OR (Functional Magnetic Resonance Imaging)) OR (fMRI)) OR (Resting-state Functional Magnetic Resonance Imaging)) OR (rs-fMRI)) OR (Neuroimaging)) OR (Regional Homogeneity)) OR (ReHo)) OR (Amplitude of Low-frequency Fluctuation)) OR (ALFF)) OR (Functional Connectivity)) OR (FC) |
| #17 | #15 OR #16 |
| #18 | #7 AND #10 AND #14 AND #17 |
| #19 | ((“1879/01/01”[Date - Publication] : “2024/12/31”[Date - Publication])) AND (#7 AND #10 AND #14 AND #17) |

**Search strategy for Embase**

| Number | Terms |
| --- | --- |
| #1 | ‘cerebrovascular accident’/exp |
| #2 | ‘accident, cerebrovascular’ OR ‘acute cerebrovascular lesion’ OR ‘acute focal cerebral vasculopathy’ OR ‘acute stroke’ OR ‘apoplectic stroke’ OR ‘apoplexia’ OR ‘apoplexy’ OR ‘blood flow disturbance, brain’ OR ‘brain accident’ OR ‘brain attack’ OR ‘brain blood flow disturbance’ OR ‘brain insult’ OR ‘brain insultus’ OR ‘brain vascular accident’ OR ‘cerebral apoplexia’ OR ‘cerebral insult’ OR ‘cerebral stroke’ OR ‘cerebral vascular accident’ OR ‘cerebral vascular insufficiency’ OR ‘cerebro vascular accident’ OR ‘cerebrovascular arrest’ OR ‘cerebrovascular failure’ OR ‘cerebrovascular injury’ OR ‘cerebrovascular insufficiency’ OR ‘cerebrovascular insult’ OR ‘cerebrum vascular accident’ OR ‘cryptogenic stroke’ OR ‘cva’ OR ‘insultus cerebralis’ OR ‘ischaemic seizure’ OR ‘ischemic seizure’ OR ‘stroke’ OR ‘thrombotic stroke’ |
| #3 | #1 OR #2 |
| #4 | ‘brain infarction’/exp |
| #5 | ‘brain cortex infarct’ OR ‘brain cortex infarction’ OR ‘brain infarct’ OR ‘cerebral cortex infarct’ OR ‘cerebral cortex infarction’ OR ‘cerebral infarct’ OR ‘cerebral infarction’ OR ‘cerebrovascular infarct’ OR ‘cerebrovascular infarction’ OR ‘cortical infarct’ OR ‘cortical infarction’ OR ‘hemisphere infarct’ OR ‘hemisphere infarction’ OR ‘hemispheric infarct’ OR ‘hemispheric infarction’ OR ‘infarction, brain’ OR ‘silent brain infarction’ |
| #6 | #4 OR #5 |
| #7 | ‘brain hemorrhage’/exp |
| #8 | ‘bleeding, corpus callosum’ OR ‘brain bleeding’ OR ‘brain haemorrhage’ OR ‘brain haemorrhage, traumatic’ OR ‘brain hemorrhage, traumatic’ OR ‘brain microhaemorrhage’ OR ‘brain microhemorrhage’ OR ‘brain stem haemorrhage, traumatic’ OR ‘brain stem hemorrhage, traumatic’ OR ‘cerebral haemorrhage’ OR ‘cerebral haemorrhage, traumatic’ OR ‘cerebral hemorrhage’ OR ‘cerebral hemorrhage, traumatic’ OR ‘cerebral microbleed’ OR ‘corpus callosum bleeding’ OR ‘corpus callosum haemorrhage’ OR ‘corpus callosum hemorrhage’ OR ‘encephalorrhagia’ OR ‘haemorrhage, brain’ OR ‘haemorrhage, intracranial’ OR ‘haemorrhagic apoplexy’ OR ‘haemorrhagic stroke’ OR ‘haemorrhagic stroke intracerebral bleeding’ OR ‘hematencephalon’ OR ‘hemorrhage, brain’ OR ‘hemorrhage, intracranial’ OR ‘hemorrhagic apoplexy’ OR ‘hemorrhagic stroke’ OR ‘hemorrhagic stroke intracerebral bleeding’ OR ‘hypertensive intracranial haemorrhage’ OR ‘hypertensive intracranial hemorrhage’ OR ‘intracerebral bleeding’ OR ‘intracerebral haemorrhage’ OR ‘intracerebral hemorrhage’ OR ‘intracortical haemorrhage’ OR ‘intracortical hemorrhage’ OR ‘intracranial bleeding’ OR ‘intracranial haemorrhage’ OR ‘intracranial haemorrhage, hypertensive’ OR ‘intracranial haemorrhage, traumatic’ OR ‘intracranial haemorrhages’ OR ‘intracranial hemorrhage’ OR ‘intracranial hemorrhage, hypertensive’ OR ‘intracranial hemorrhage, traumatic’ OR ‘intracranial hemorrhages’ OR ‘intraventricular haemorrhage’ OR ‘intraventricular hemorrhage’ OR ‘periventricular haemorrhage’ OR ‘periventricular hemorrhage’ OR ‘posterior fossa haemorrhage’ OR ‘posterior fossa hemorrhage’ OR ‘traumatic brain haemorrhage’ OR ‘traumatic brain hemorrhage’ OR ‘traumatic brain stem haemorrhage’ OR ‘traumatic brain stem hemorrhage’ OR ‘traumatic cerebral haemorrhage’ OR ‘traumatic cerebral hemorrhage’ OR ‘traumatic intracranial haemorrhage’ OR ‘traumatic intracranial hemorrhage’ |
| #9 | #7 OR #8 |
| #10 | #3 OR #6 OR #9 |
| #11 | ‘transcranial magnetic stimulation’/exp |
| #12 | ‘magnetic stimulation, transcranial’ OR ‘stimulation, transcranial magnetic’ OR ‘transcranial magnetic stimulation, single pulse’ OR ‘transcranial magnetic stimulation, paired pulse’ OR ‘transcranial magnetic stimulation, repetitive’ OR ‘rtms’ OR ‘hf-rtms’ OR ‘lf-rtms’ OR ‘high frequency-rtms’ OR ‘low frequency-rtms’ OR ‘intermittent theta burst stimulation’ OR ‘itbs’ OR ‘continuous theta burst stimulation’ OR ‘ctbs’ OR ‘non-invasive’ |
| #13 | #11 OR #12 |
| #14 | ‘cognitive defect’/exp |
| #15 | ‘cognition’ OR ‘cognitive dysfunction’ OR ‘cognitive impairment’ OR ‘memory’ OR ‘attention’ OR ‘language’ OR ‘executive function’ OR ‘visuospatial function’ |
| #16 | #14 OR #15 |
| #17 | ‘nuclear magnetic resonance imaging’/exp |
| #18 | ‘magnetic resonance imaging’ OR ‘functional magnetic resonance imaging’ OR ‘fmri’ OR ‘resting-state functional magnetic resonance imaging’ OR ‘rs-fmri’ OR ‘neuroimaging’ OR ‘regional homogeneity’ OR ‘reho’ OR ‘amplitude of low-frequency fluctuation’ OR ‘alff’ OR ‘functional connectivity’ OR ‘fc’ |
| #19 | #17 OR #18 |
| #20 | #10 AND #13 AND #16 AND #19 |
| #21 | #10 AND #13 AND #16 AND #19 AND [<1966-2024]/py |

**Search strategy for Cochrane Library**

| Number | Terms |
| --- | --- |
| #1 | MeSH descriptor: [Stroke] explode all trees |
| #2 | (Cerebral Strokes OR Cerebrovascular Accident OR Cerebral Stroke OR Strokes, Cerebral OR Cerebrovascular Stroke OR Strokes, Cerebrovascular OR Apoplexy OR Stroke, Cerebral OR Cerebrovascular Accident OR Strokes OR Vascular Accident, Brain OR Stroke, Cerebrovascular OR Cerebrovascular Strokes OR CVA OR Brain Vascular Accident OR Apoplexy, Cerebrovascular OR Brain Vascular Accidents OR Cerebrovascular Apoplexy OR Cerebrovascular Accidents OR Cerebrovascular Accidents, Acute OR Strokes, Acute OR Acute Stroke OR Acute Cerebrovascular Accidents OR Stroke, Acute OR Cerebrovascular Accident, Acute OR Acute Cerebrovascular Accident OR Acute Strokes) |
| #3 | #1 OR #2 |
| #4 | MeSH descriptor: [Cerebral Infarction] explode all trees |
| #5 | (Cerebral, Left Hemisphere, Infarction OR Left Hemisphere, Infarction, Cerebral OR Cerebral Infarction, Left Hemisphere OR Left Hemisphere, Cerebral Infarction OR Infarction, Left Hemisphere, Cerebral OR Infarction, Cerebral, Left Hemisphere OR Posterior Choroidal Artery Infarction OR Infarctions, Subcortical OR Subcortical Infarctions OR Infarction, Subcortical OR Subcortical Infarction OR Anterior Choroidal Artery Infarction OR Infarction, Right Hemisphere, Cerebral OR Cerebral Infarction, Right Hemisphere OR Cerebral, Right Hemisphere, Infarction OR Infarction, Cerebral, Right Hemisphere OR Right Hemisphere, Infarction, Cerebral OR Right Hemisphere, Cerebral Infarction OR Infarctions, Cerebral OR Cerebral Infarct OR Cerebral Infarctions OR Infarcts, Cerebral OR Cerebral Infarcts OR Infarction, Cerebral OR Infarct, Cerebral) |
| #6 | #4 OR #5 |
| #7 | MeSH descriptor: [Cerebral Hemorrhage] explode all trees |
| #8 | (Cerebral Brain Hemorrhages OR Cerebral Parenchymal Hemorrhages OR Hemorrhage, Cerebral OR Cerebral Brain Hemorrhage OR Brain Hemorrhages, Cerebral OR Cerebral Hemorrhages OR Brain Hemorrhage, Cerebral OR Parenchymal Hemorrhages, Cerebral OR Intracerebral Hemorrhage OR Cerebrum Hemorrhages OR Hemorrhage, Cerebral Parenchymal OR Hemorrhages, Cerebral Parenchymal OR Hemorrhage, Cerebral Brain OR Hemorrhage, Cerebrum OR Hemorrhage, Intracerebral OR Hemorrhages, Cerebral OR Cerebrum Hemorrhage OR Hemorrhages, Cerebral Brain OR Cerebral Parenchymal Hemorrhage OR Parenchymal Hemorrhage, Cerebral OR Intracerebral Hemorrhages OR Hemorrhages, Intracerebral OR Hemorrhages, Cerebrum) |
| #9 | #7 OR #8 |
| #10 | #3 OR #6 OR #9 |
| #11 | MeSH descriptor: [Transcranial Magnetic Stimulation] explode all trees |
| #12 | (Magnetic Stimulation, Transcranial OR Magnetic Stimulations, Transcranial OR Stimulation, Transcranial Magnetic OR Stimulations, Transcranial Magnetic OR Transcranial Magnetic Stimulations OR Transcranial Magnetic Stimulation, Single Pulse OR Transcranial Magnetic Stimulation, Paired Pulse OR Transcranial Magnetic Stimulation, Repetitive OR rTMS OR HF-rTMS OR LF-rTMS OR High Frequency-rTMS OR Low Frequency-rTMS OR Intermittent Theta Burst Stimulation OR iTBS OR Continuous Theta Burst Stimulation OR cTBS OR Non-invasive) |
| #13 | #11 OR #12 |
| #14 | MeSH descriptor: [Cognition] explode all trees |
| #15 | MeSH descriptor: [Cognitive Dysfunction] explode all trees |
| #16 | (Cognition OR Cognitive Dysfunction OR Cognitive Impairment OR Memory OR Attention OR Language OR Executive Function OR Visuospatial Function) |
| #17 | #14 OR #15 OR #16 |
| #18 | MeSH descriptor: [Magnetic Resonance Imaging] explode all trees |
| #19 | (Magnetic Resonance Imaging OR Functional Magnetic Resonance Imaging OR fMRI OR Resting-state Functional Magnetic Resonance Imaging OR rs-fMRI OR Neuroimaging OR Regional Homogeneity OR ReHo OR Amplitude of Low-frequency Fluctuation OR ALFF OR Functional Connectivity OR FC) |
| #20 | #18 OR #19 |
| #21 | #10 AND #13 AND #17 AND #20 |
| #22 | 筛选日期：01/01/1947- 31/12/2024 |

**Search strategy for Web of Science**

| Number | Terms |
| --- | --- |
| #1 | **ALL=(Stroke OR Cerebral Strokes OR Cerebrovascular Accident OR Cerebral Stroke OR Strokes, Cerebral OR Cerebrovascular Stroke OR Cerebral Infarction OR Cerebral Hemorrhage)** |
| #2 | **ALL=(Transcranial Magnetic Stimulation OR Magnetic Stimulation, Transcranial OR Magnetic Stimulations, Transcranial OR Stimulation, Transcranial Magnetic OR Stimulations, Transcranial Magnetic OR Transcranial Magnetic Stimulations OR Transcranial Magnetic Stimulation, Single Pulse OR Transcranial Magnetic Stimulation, Paired Pulse OR Transcranial Magnetic Stimulation, Repetitive OR rTMS OR HF-rTMS OR LF-rTMS OR High Frequency-rTMS OR Low Frequency-rTMS OR Intermittent Theta Burst Stimulation OR iTBS OR Continuous Theta Burst Stimulation OR cTBS** OR Non-invasive**)** |
| #3 | **ALL=(Cognition OR Cognitive Dysfunction OR Cognitive Impairment OR Memory OR Attention OR Language OR Executive Function OR Visuospatial Function)** |
| #4 | **ALL=(Magnetic Resonance Imaging OR Functional Magnetic Resonance Imaging OR fMRI OR Resting-state Functional Magnetic Resonance Imaging OR rs-fMRI OR Neuroimaging OR Regional Homogeneity OR ReHo OR Amplitude of Low-frequency Fluctuation OR ALFF OR Functional Connectivity OR FC)** |
| #5 | **#1 AND #2 AND #3 AND #4** |

**CBM检索式：**

(((“磁共振成像”[全部字段] OR “功能磁共振成像”[全部字段] OR “静息态功能磁共振成像”[全部字段] OR “fMRI”[全部字段] OR “rs-fMRI”[全部字段] OR “区域同质性”[全部字段] OR “局部一致性”[全部字段] OR “ReHo”[全部字段] OR “低频振幅”[全部字段] OR “ALFF”[全部字段] OR “功能连接”[全部字段] OR “FC”[全部字段]) OR (“磁共振成像”[不加权:扩展])) AND ((“认知功能”[全部字段] OR “认知障碍”[全部字段] OR “认知功能障碍”[全部字段] OR “记忆”[全部字段] OR “注意”[全部字段] OR “语言”[全部字段] OR “执行功能”[全部字段] OR “视空间功能”[全部字段]) OR (“认知功能障碍”[不加权:扩展]) OR (“认知障碍”[不加权:扩展]) OR (“认知”[不加权:扩展])) AND ((“重复经颅磁刺激”[全部字段] OR “低频rTMS”[全部字段] OR “高频rTMS”[全部字段] OR “间断性theta节律刺激”[全部字段] OR “连续性theta节律刺激”[全部字段] OR “iTBS”[全部字段] OR “cTBS”[全部字段] OR “rTMS”[全部字段] OR “非侵入性脑刺激技术”[全部字段] OR “无创性脑刺激技术”[全部字段]) OR (“经颅磁刺激”[不加权:扩展])) AND ((“脑卒中”[全部字段] OR “中风”[全部字段] OR “脑血管意外”[全部字段] OR “脑出血”[全部字段] OR “脑梗死”[全部字段] OR “缺血性卒中”[全部字段] OR “出血性卒中”[全部字段]) OR (“脑梗死”[不加权:扩展]) OR (“脑出血”[不加权:扩展]) OR (“脑缺血”[不加权:扩展]) OR (“中风”[不加权:扩展]) OR (“卒中”[不加权:扩展]))) AND -2024[日期]

**CNKI检索式：**

| #1 | 主题：脑卒中 + 脑梗死 + 脑出血 + 脑血管意外 + 中风 + 缺血性卒中 + 出血性卒中  篇关摘：脑卒中 + 脑梗死 + 脑出血 + 脑血管意外 + 中风 + 缺血性卒中 + 出血性卒中  关键词：脑卒中 + 脑梗死 + 脑出血 + 脑血管意外 + 中风 + 缺血性卒中 + 出血性卒中  篇名：脑卒中 + 脑梗死 + 脑出血 + 脑血管意外 + 中风 + 缺血性卒中 + 出血性卒中 | 精确  同义词扩展 |
| --- | --- | --- |
| #2 | 结果中检索  主题：经颅磁刺激 + 重复性经颅磁刺激 + 间断性theta节律刺激 + 连续性theta节律刺激 + 低频rTMS + 高频rTMS +非侵入性脑刺激技术 + 无创性脑刺激技术  篇关摘：经颅磁刺激 + 重复性经颅磁刺激 + 间断性theta节律刺激 + 连续性theta节律刺激 + 低频rTMS + 高频rTMS + 非侵入性脑刺激技术 + 无创性脑刺激技术  关键词：经颅磁刺激 + 重复性经颅磁刺激 + 间断性theta节律刺激 + 连续性theta节律刺激 + 低频rTMS + 高频rTMS + 非侵入性脑刺激技术 + 无创性脑刺激技术  篇名：经颅磁刺激 + 重复性经颅磁刺激 + 间断性theta节律刺激 + 连续性theta节律刺激 + 低频rTMS + 高频rTMS +非侵入性脑刺激技术 + 无创性脑刺激技术 | 精确  同义词扩展 |
| #3 | 结果中检索  主题：认知功能 + 认知障碍 + 认知功能障碍 + 记忆 + 注意 + 语言 + 执行功能 + 视空间功能  篇关摘：认知功能 + 认知障碍 + 认知功能障碍 + 记忆 + 注意 + 语言 + 执行功能 + 视空间功能  关键词：认知功能 + 认知障碍 + 认知功能障碍 + 记忆 + 注意 + 语言 + 执行功能 + 视空间功能  篇名：认知功能 + 认知障碍 + 认知功能障碍 + 记忆 + 注意 + 语言 + 执行功能 + 视空间功能 | 精确  同义词扩展 |
| #4 | 结果中检索  主题：磁共振成像 + 功能磁共振成像 + 静息态功能磁共振成像 + fMRI + rs-fMRI + 区域同质性 + 局部一致性 + ReHo + 低频振幅 + ALFF + 功能连接 + FC  篇关摘：磁共振成像 + 功能磁共振成像 + 静息态功能磁共振成像 + fMRI + rs-fMRI + 区域同质性 + 局部一致性 + ReHo + 低频振幅 + ALFF + 功能连接 + FC  关键词：磁共振成像 + 功能磁共振成像 + 静息态功能磁共振成像 + fMRI + rs-fMRI + 区域同质性 + 局部一致性 + ReHo + 低频振幅 + ALFF + 功能连接 + FC  篇名：磁共振成像 + 功能磁共振成像 + 静息态功能磁共振成像 + fMRI + rs-fMRI + 区域同质性 + 局部一致性 + ReHo + 低频振幅 + ALFF + 功能连接 + FC | 精确  同义词扩展 |
| #5 | 结果中检索：设置截止日期2024年12月31日 |  |

**万方检索式：**

(全部:(脑卒中 or 中风 or 脑血管意外 or 脑梗死 or 脑出血 or 缺血性卒中 or 出血性卒中) and 全部:(经颅磁刺激 or 重复性经颅磁刺激 or 间断性theta节律刺激 or 连续性theta节律刺激 or 低频rTMS or 高频rTMS or 非侵入性脑刺激技术 or 无创性脑刺激技术) and 全部:(认知功能 or 认知障碍 or 认知功能障碍 or 记忆 or 注意 or 语言 or 执行功能 or 视空间功能) and 全部:(磁共振成像 or 功能磁共振成像 or 静息态功能磁共振成像 or fMRI or rs-fMRI or 区域同质性 or 局部一致性 or ReHo or 低频振幅 or ALFF or 功能连接 or FC)) and 出版时间:[* TO 2024-12-31}

**维普检索式：**

检索操作如下：

任意字段：脑卒中 or 脑梗死 or 脑出血 or 脑血管意外 or 中风 or 缺血性卒中 or 出血性卒中 【模糊】

任意字段：经颅磁刺激 or 重复性经颅磁刺激 or 间断性theta节律刺激 or 连续性theta节律刺激 or 低频rTMS or 高频rTMS or 非侵入性脑刺激技术 or 无创性脑刺激技术 【模糊】

任意字段：认知功能 or 认知障碍 or 认知功能障碍 or 记忆 or 注意 or 语言 or 执行功能 or 视空间功能 【模糊】

任意字段：磁共振成像 or 功能磁共振成像 or 静息态功能磁共振成像 or fMRI or rs-fMRI or 区域同质性 or 局部一致性 or ReHo or 低频振幅 or ALFF or 功能连接 or FC 【模糊】

时间：收录-2024年
