# Supplementary material for: The Use of Repetitive Transcranial Magnetic Stimulation to Improve Cognitive Impairment in Patients With Stroke Based on rs-fMRI Findings: Protocol for a Meta-Analysis
Source: JMIR Res Protoc. 2025 Oct 2;14:e77931. doi: 10.2196/77931 (PMC12531584; doi:10.2196/77931)
Supplement: Multimedia Appendix 3 [file resprot_v14i1e77931_app3.docx]

**Multimedia Appendix 3. Criteria for objective assessment of the methodological quality of individual studies**

| **Category 1: Sample characteristics (10)** |
| --- |
| 1. Patients were evaluated with specific standardised diagnostic criteria (1) |
| 1. Important demographic data (age and gender) were reported with mean (or median) and standard deviations (or range)) (2) |
| 1. Healthy comparison subjects were evaluated to exclude psychiatric and medical illnesses and demographic data was reported (1) |
| 1. Important clinical variables (e.g. illness duration, onset time, medication status, pain scores) were reported with mean (or median) and standard deviations (or range)) (4) |
| 1. Sample size per group > 10 (2) |
| **Category 2: Methodology and reporting (10)** |
| 1. Whole brain analysis was automated with no a-priori regional selection (3) |
| 1. Magnet strength at least 1.5T (1) |
| 1. At least 5 minutes of resting state acquisition (1) |
| 1. Whole brain coverage of resting scans (1) |
| 1. The acquisition and preprocessing techniques were clearly described so that they could be reproduced (1) |
| 1. Coordinates reported in a standard space (1) |
| 1. Significant results are reported after correction for multiple testing using a standard statistical procedure (FDR, FWE or permutation-based methods) (1) |
| 1. Conclusions were consistent with the results obtained and the limitations were discussed (1) |
